# Primary pure angiosarcoma of the testis: a vanishingly rare malignancy. Case report and literature review

**DOI:** 10.1186/s12894-020-00747-7

**Published:** 2020-10-31

**Authors:** J. Walravens-Evans, M. Yao, S. Grannò, D. Arul, S. Chitale

**Affiliations:** 1 Kingston Hospital NHS Foundation Trust, Galsworthy Road, Surrey, KT2 7QB UK; 2 Department of Urology, Kent and Canterbury Hospital, Ethelbert Road, Canterbury, CT1 3NG UK; 3 UCL School of Life and Medical Sciences, Gower St, London, WC1E 6BT UK; 4 Department of Pathology, Whittington Hospital, Magdala Avenue, London, N19 5NF UK; 5 Department of Urology, Whittington Hospital, Magdala Avenue, London, N19 5NF UK

**Keywords:** Angiosarcoma, Testicular malignancy, Immunohistochemistry, Cisplatin, Radiotherapy, Embryonal carcinoma

## Abstract

**Background:**

Primary pure angiosarcoma of the testis is an exceptionally rare testicular malignancy, which is poorly understood. We present the fifth and youngest case in the current medical literature. Additionally, all cases of angiosarcoma of the testicle, both occurring with associated germ cell tumour and without, were compared in an extended tabular format.

**Case presentation:**

A 56-year old man presented with unilateral scrotal pain, swelling and erythema. Ultrasonography revealed two testicular lesions with a high suspicion of malignancy but serum tumour markers were negative. A radical orchidectomy was performed with clear surgical margins. Diagnosis of primary pure angiosarcoma of the testis was confirmed on subsequent histopathology.

**Conclusions:**

Primary pure angiosarcoma is a rare testicular neoplasm. We present the fifth case in the literature. Clinical and radiological features are non-specific. The diagnosis is purely histological, with the pathologist choosing immunohistochemistry based on abnormal morphology. Local invasiveness is variable but metastatic sites are typical for extra-gonadal angiosarcomas. Primary pure testicular angiosarcoma diagnosis confers a relatively better prognosis compared to angiosarcoma arising in the context of a testicular germ cell tumour. While extra-gonadal angiosarcomas are associated with high rates of local recurrence following resection, in all cases of testicular angiosarcoma there were no local recurrences following radical orchidectomy. Surgical resection remains the most effective treatment for both subtypes of testicular angiosarcoma.

## Background

Angiosarcomas are an uncommon group of malignancies that arise from vascular endothelial cells and generally carry a poor prognosis. Reported five-year survival rate is 35% in non-metastatic disease [1]. Incidence is highest in the sixth and seventh decades of life, although angiosarcoma can develop at any age [2]. Aetiological factors known to give rise to angiosarcoma include radio- or chemotherapy, vinyl chloride, arsenic and thorium dioxide [2, 3]. These malignancies behave aggressively, with extensive local invasion, early and widespread metastases and high rates of local recurrence following resection [2, 3]. Metastasis is thought to occur via haematogenous spread [4]. The most frequent sites of metastasis are the lungs, liver, bones and lymph nodes [4]. In most cases of angiosarcoma, extensive metastatic disease is present at the time of diagnosis [4]. Surgical resection, where possible, comprises the mainstay of treatment. Angiosarcomas are relatively unresponsive to chemotherapy and radiotherapy [5–7]. Angiosarcoma arising as a primary malignancy in the testis (PPAS) is extremely rare.

Germ cell tumours (GCT) are the most common testicular neoplasm, which most frequently comprises teratoma or seminoma [8, 9]. GCTs affect young men, the majority of cases occur between the ages of 20–39 years [10, 11]. Two to four percent of teratomatous elements within GCTs undergo malignant transformation [6, 12, 13], and sarcomatous transformation predominates over carcinoma [7, 14]. Transformation of testicular GCT to angiosarcoma is extremely rare, with eight cases reported in the literature to date. Once angiosarcoma arises, it can metastasise with, or independently, of other tumour cell types and its behaviour is usually aggressive [15].

For the purposes of this paper, the following subtypes of angiosarcoma are henceforth referred to as: extra-testicular soft tissue angiosarcomas (extra-gonadal), and two gonadal subtypes: primary pure angiosarcoma of the testis (PPAS), and angiosarcoma as a component of a testicular germ cell tumour (AS-GCT). In the latter, the angiosarcoma component might arise either within the testicular primary or within a germ cell tumour metastasis.

PPAS and AS-GCT are extremely rare, thus little is known about these conditions and further work is required. Here, we describe the fifth and youngest case of PPAS. Following, we summarise all previous cases of PPAS [16–19] and AS-GCT [7, 13, 15, 20–23] via an extended tabular approach.

## Case presentation

We report our case of a 56-year-old man of Bulgarian origin working in the UK, who initially presented to primary care with a four-day history of right scrotal swelling, redness and pain, with a palpable abnormal testicular mass on examination. The patient had no past medical or family history of note. He was a 30-pack year smoker, with no occupational exposure, nor any history of previous chemo- or radio-therapy. He was treated with oral antibiotics for presumptive clinical diagnosis of epididymo-orchitis and an urgent ultrasound scan was arranged in secondary care. All blood tests were normal, including a full blood count and differential, urea and electrolytes, β-HCG, lactate dehydrogenase (LDH), and alpha-fetoprotein (AFP).

**Fig. 1:**
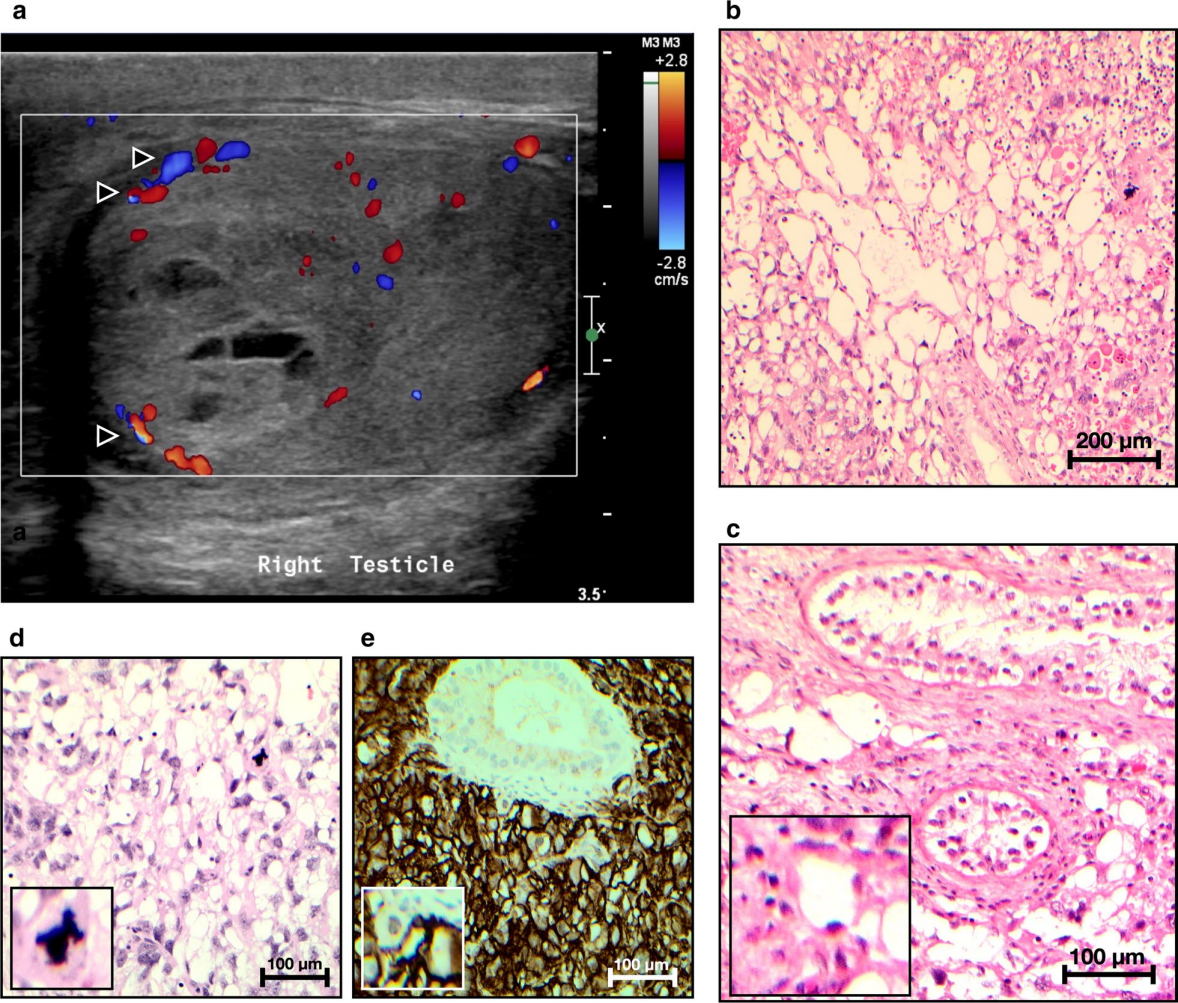
Micro- and Macroscopic appearance of this case of PPAS. **a** Doppler ultrasound scan of the patient’s right testicle, demonstrating one of the suspicious lesions (white square), with areas of vascularity that surrounds the lesion but not evident within it (black arrowheads with white border). **b** Hematoxylin and eosin stain of primary testicular AS at 10 × magnification. **c** Hematoxylin and eosin stain of PPAS at 20 × magnification, demonstrating a seminiferous tubule and several malignant cells infiltrating in and around the tubules. **d** Hematoxylin and eosin stain of PPAS at 20 × magnification, demonstrating markedly atypical, plump endothelial cells with atypical mitoses. **e** CD31 staining at 20 × magnification, demonstrating strong and diffuse CD31 immunopositivity

Ultrasonography demonstrated two discrete, heterogenous, hypoechoic lesions within the parenchyma of the right testis, with some cystic changes. The larger lesion measured 2 cm in diameter. There was no evidence of extension beyond the testicular tunica albuginea. Angiosarcomas typically appear highly vascular on ultrasonography, however in this case was there was no evidence of internal vascularity (Fig. 1a). Bilateral simple epididymal cysts were noted, as well as a small volume hydrocele of the left testis.

In the context of partially treated epididymo-orchitis, our differential diagnoses at that time included intra-testicular abscess and primary testicular neoplasm, potentially a teratoma. The case was discussed at local multi-disciplinary team (MDT) meeting and a right radical orchidectomy as a definitive therapeutic procedure was performed.

Pathological examination revealed the right testis to measure 50 × 40 × 40 mm (average male 50 × 20 × 30 mm), and the spermatic cord measured 45 × 20 × 25 mm. This gives an estimated testicular volume of 41.6 ml, where a normal range is 12–30 ml [24]. On gross inspection, two discrete, adjacent lesions distorted the testicular parenchyma. The first lesion was a cream-coloured tumour that measured 17 × 19 × 17 mm. The second lesion comprised an irregular haemorrhagic area with an ill-defined edge that measured 20 × 20 × 20 mm. The surrounding parenchyma appeared macroscopically normal. Neither lesion appeared to infiltrate the tunica or hilum.

Microscopically, both lesions displayed similar appearances (Fig. 1b–e). Each comprised sheets of cells, mainly with a lace-like pattern of infiltration, although in places solid sheets of cells were visualised. The cells exhibited markedly pleomorphic nuclei with prominent nucleoli. The cytoplasm contained eosinophilic granules of variable sizes. Large areas of haemorrhage and focal necrosis were noted. Separate to these nodules, tumour was noted to infiltrate between seminiferous tubules (Fig. 1c) and into the stroma of the rete testis, but not into the hilum. Resected surgical margins were negative.

Immunohistochemical analysis was performed. The lesions were diffusely and strongly positive for vascular markers, CD31 and CD34. The markers looking for potential background germ cell tumour (AFP, Oct3/4, Glypican 3, Beta-HCG, CD30 and CD117) were negative. The following antibodies were also negative: EMA, S100, CAM5.2, MNF116, BerEp4 and calretinin.

In light of these findings, the final histopathological diagnosis was given as high-grade angiosarcoma ie PPAS. The patient had a re-staging CT scan three months post operatively due to lower back pain, which demonstrated no evidence of recurrence or metastatic disease. Subsequent follow up continued with active surveillance at a tertiary centre. The patient remained well at review six months after initial presentation.

## Discussion and conclusions

We have reported the fifth case of primary pure angiosarcoma (PPAS) of the testis. The four existing cases of PPAS, plus the index case, are summarised in Tables 1 and 2 [16–19]. The eight previous cases of AS-GCT are summarised in Tables 3 and 4 [7, 13, 15, 20–23].

**Table 1:** Clinical and pathological features of all known cases of PPAS

Case	Age (years)	Diagnosis	Presentation		Investigations		Macroscopic features of testicular lesion		
			Symptoms (duration)	Patient noticed lump? (duration)	Bloods	Testicular ultrasound	Site	Appearance	Size (mm)
Current case	56	PPAS	Testicular pain, swelling, redness (4 days)	No	Normal	Two lesions Heterogenous, hypoechoic, some cysts, no vascularity	Parenchyma, rete testis, tunica	*A:* cream-coloured, Solid *B:* Haemorrhagic, infiltrative	*A:* 17 × 17 × 19 *B:* 20 × 20 × 20
Piotrowski et al. [19]	58	PPAS	Hip pain, back pain (3 months)	No	Normal	Two lesions	*A:* Parenchyma, epididymis *B:* Spermatic cord	*N/A*	*A:* 120 × 68 × 68 *B:* 35 × 24 × 20
Jain et al. [18]	63	PPAS	Testicular enlargement (8 months), preceding testicular firmness (10 years)	Yes (10 years)	Normal	Solid lesion	Parenchyma replaced by tumour	Haemorrhagic, necrotic, focal solid white	110 × 80 × 70
Armah et al. [17]	80	PPAS	Painless lump (2 months), hydrocoele (7 years)	Yes (2 months)	*N/A*	*N/A*	Parenchyma, epididymis	Haemorrhagic, solid	30
Mašera et al. [16]	74	PPAS, epithelioid	Fever unknown cause (3 weeks)	No	CRP↑ ESR↑	Transonic, vascularity	Parenchyma	Haemorrhagic, brown-white, infiltrative	17

**Table 2:** Therapeutic strategies and outcomes for all known cases of PPAS

Case	Testicular lesion		Metastases	Outcome
	Treatment (margins)	Local recurrence		
Current case	Orchidectomy (clear)	No	No	Alive and well after 6 months
Piotrowski et al. [19]			*At presentation:* Bones, lungs, right retroperitoneum, right pelvic LN’s	Died after 6 months
Jain et al. [18]			*3 months after presentation:* Widespread bones	Died after 3 months
Armah et al. [17]			No	Alive and well after 20 months
Mašera et al. [16],			No	Died after 1 month from stroke

**Table 3:** Clinical and pathological features of all known cases of AS-GCT

Case	Age (years)	Diagnosis	Presentation		Testicular lesion				
			Symptoms (duration)	Bloods	Diagnosis	Site	Size (mm)	Definitive treatment (margins)	Local recurrence
*Primary angiosarcoma in germ cell tumour*									
Malagón et al. [15]	25	AS-GCT	*N/A*	*N/A*	Teratoma, yolk sac tumour, angiosarcoma	*N/A*	*N/A*	Orchidectomy (*N/A*)	*N/A*
Malagón et al. [15],	35	AS-GCT	*N/A*	*N/A*	Teratoma, yolk sac tumour	*N/A*	*N/A*	Orchidectomy (*N/A*)	*N/A*
Sahoo et al., [23]	23	AS-GCT, epithelioid	Right flank pain, back pain	Normal	Teratoma	Parenchyma	65 × 60 × 55	Orchidectomy (clear)	No
Steele et al. [22]	24	AS-GCT, epithelioid	Left testis mass (6 months)	Normal	Teratoma, epithelioid angiosarcoma	Parenchyma, rete testis, epididymis, spermatic cord	80 × 70	Orchidectomy (clear)	No
Hughes et al. [20]	16	AS-GCT	Right testis mass (1 months)	Normal	Teratoma, angiosarcoma	Parenchyma	90 × 70 × 65	Orchidectomy (clear) post-op RT	No
*Therapy-related angiosarcoma in germ cell tumour*									
Idrees et al. [7]	38	AS-GCT	Abdominal pain	AFP 8320	Teratoma, yolk sac tumour, seminoma	Parenchyma, epididymis, para-testicular soft tissue	*N/A*	Orchidectomy (clear)	No
Lee et al. [21]	35	AS-GCT, epithelioid	*N/A*	*N/A*	Seminoma	*N/A*	80	Orchidectomy (clear)	No
Ulbright et al. [6]	17	AS-GCT	Testis mass, back pain (1 years)	*N/A*	Teratoma	*N/A*	*N/A*	Orchidectomy (*N/A*)	No

**Table 4:** Disease progression, treatment and misdiagnosis of all known cases of AS-GCT

Case	Diagnosis				Treatment after angiosarcoma developed		Outcome at follow-up (time interval)
	Primary testicular diagnosis	Metastatic sites (tumour diagnosis)	Risk factors for angiosarcoma	Misdiagnosis	Chemotherapy	Surgery	
*Primary angiosarcoma in germ cell tumour*							
Malagón et al. [15]	Mature teratoma, yolk sac tumour, angiosarcoma	Retroperitoneum (N/A)	No	No	Cisplatin, Cyclophosphamide and Adriamycin	*N/A*	Alive with mets (8 months)
Malagón et al. [15]	Mature teratoma, angiosarcoma	Lungs, LNs (MT, AS)	No	No	Cisplatin, Cyclophosphamide and Adriamycin	*N/A*	Alive with mets (72 months)
Sahoo et al. [23]	Mature teratoma	Retroperitoneum (MT, AS), liver (AS), lungs, pleura (AS), retroperitoneal LNs (AS)	No	Yes, met: MT with epithelioid AS diagnosed as MT with embryonal carcinoma	Thalidomide	Resection, LND	Alive with mets (22 months)
					Cisplatin and Gemcitabine		
Steele et al. [22]	Mature teratoma, epithelioid angiosarcoma	Lungs (AS), renal hilum (AS), pre-aortic, interaortocaval and renal hilar LNs (AS)	No	Yes, primary: MT with epithelioid AS diagnosed as MT with embryonal carcinoma	Cisplatin, Etoposide and Bleomycin	Resection, RPLND	Alive with recurrent mets
					Ifosfamide and Doxorubicin		
Hughes et al. [20]	Mature teratoma, angiosarcoma	No	No	No	No	No	Alive with no mets (9 months)
*Therapy-related angiosarcoma in germ cell tumour*							
Idrees et al. [7]	Mature teratoma, seminoma, yolk sac tumour	Lungs (MT), retroperitoneum (MT), para-iliac, para-caval and retrocrural LNs (MT), LNs near thoracic duct (MT), posterior mediastinum (AS)	Cisplatin, Etoposide and Bleomycin (40 months)	No	No	6 Resections over 4 years	Alive with no mets (58 months)
			Vinblastine, Ifosfamide and Cisplatin (40 months)				
Lee et al. [21]	Seminoma	Paravertebral (AS), lungs (AS), thorax (AS), liver (AS)	RT (10 years)	No	MTX and leucovorin	Resections	Died (13 months after mets)
			Cisplatin, Vinblastine and Bleomycin (13 months)				
Ulbright et al. [6]	Mature teratoma	Retroperitoneum (MT), lungs, liver (AS), kidneys (AS), adrenals (AS), spleen (AS)	RT (5 years)	No	No	No	Died (5 years)
			Cisplatin, Vinblastine and Bleomycin (5 years)				

PPAS tends to occur in elderly patients (median 63 years, 56–80, *n* = 5), whereas AS-GCT affects younger men (median 27 years, 16–38, *n* = 8). The aetiology of all four previous cases of PPAS, plus the index case presented, remains unknown. Previous radiation therapy and chemotherapy are known causative factors in the pathogenesis of extra-gonadal angiosarcomas [2, 3]. Indeed, angiosarcoma first developed in three of the eight cases of AS-GCT following the use of platinum-based chemotherapy regimens and/or radiotherapy to treat primary testicular germ cell tumours. It has been hypothesised that these systemic therapies might have triggered clonal progression to angiosarcoma as well as hastening metastasic disease due to selective eradication of less aggressive germ cell tumour cell types [5, 7, 13, 21]. However, while some of the testicular primaries were extensively sampled, it is possible that components of angiosarcoma had already been present within the primary germ cell tumours prior to systemic treatment, but were missed. In contrast, the remaining five cases of AS-GCT did not occur in association with any known risk factor for extra-gonadal angiosarcoma [15, 20, 22, 23]. Angiosarcoma was identified as a component of teratomatous germ cell tumours prior to the administration of systemic therapies. In these cases, the angiosarcoma components were postulated to occur as a consequence of malignant transformation within teratomatous foci [15, 20, 22, 23].

In comparison, none of the four previous cases of PPAS, nor the index case, were exposed to any identifiable risk factor for angiosarcoma. Malignant transformation of a teratoma is unlikely to explain the pathogenesis of PPAS, because no teratomatous components were identified on microscopic or immunohistochemical evaluation of any case. Furthermore, malignant teratomas seldom occur in the elderly [25]. Therefore, the etiology of PPAS remains unclear.

Clinical and radiological features prior to definitive surgery (radical orchidectomy) are *not* diagnostic of PPAS [20, 22, 23]. On ultrasonography, the index case did not display the internal vascularity that is regarded as a characteristic feature of extra-gonadal angiosarcomas (Fig. 1a). This is in contrast to a previous case of PPAS which demonstrated prominent internal vascularity on ultrasound [16]. Diagnosis is made following radical orchidectomy, by microscopic identification of characteristic morphologic features and confirmation employing immunohistochemistry. Nevertheless, this approach should be undertaken cautiously, since AS-GCT and PPAS are similar in both morphology and immunophenotype [4]. AS-GCT, however, demonstrates background germ cell tumour, whereas PPAS appears to arise de novo. Histologically, angiosarcoma comprises two main subtypes: classic (spindled cells) and epithelioid (plump epithelial-like cells). These malignancies also display variable architecture (solid or vasoformative) [4]. Epithelioid angiosarcomas are often macro- and microscopically similar to embryonal carcinomas [20, 22, 23]. They are distinguishable by subtle differences on microscopy and stain differently upon immunohistochemistry, however [20, 22, 23].

Initial misdiagnosis as embryonal carcinoma with teratoma occurred upon macroscopic and microscopic evaluation of two surgical AS-GCT specimens [22, 23]. Since embryonal carcinoma and epithelioid angiosarcoma often exhibit similar morphological characteristics, [22, 23] we highlight the importance of considering the diagnosis of angiosarcoma when examining unusual testicular malignancies, with use of appropriate immunohistochemistry if necessary. Accurate diagnosis of testicular angiosarcoma is of extreme clinical relevance. Missing this diagnosis indeed has a significant detrimental effect on patient management and prognosis [22, 23].

Local invasiveness was variable between cases of PPAS (Table 1). In the index case there was no evidence of local invasion, with two discrete lesions confined within the rete testis and tunica albuginea. In the case of Piotrowski et al*.* [19] one tumour minimally extended into ipsilateral proximal spermatic cord and another into the epididymis. In the cases of Jain et al*.* [18] and Masera et al*.* [16] there was no local invasion. In the former case the entire testicular parenchyma was replaced by tumour and the patient complained of testicular firmness dating back ten years. In Armah et al*.* [17]*,* there was invasion into the epididymis. We found no apparent explanation for the discrepancy in extent of local invasion between cases of PPAS.

The contralateral testis was not affected by metastatic disease in any of the cases of PPAS or AS-GCT. Metastatic sites of the PPAS and AS-GCT cases were similar to those of extra-gonadal angiosarcoma (Tables 2 and 4) [4].

Although extra-gonadal soft tissue angiosarcomas are associated with high rates of local recurrence [4], none of the thirteen testicular cases (index case included) experienced local recurrence following radical orchidectomy. This could be a consequence of negative surgical margins due to resection high up at the level of the spermatic cord, which enabled total oncological clearance via radical orchidectomy.

All cases of PPAS were treated using radical orchidectomy only (Table 2). In contrast, five of the eight cases of AS-GCT were treated using surgery with adjuvant chemotherapy after the angiosarcomatous component arose (Table 4). However, none of the chemotherapy regimens that were employed to treat AS-GCT eradicated disease [7, 13, 15, 21–23]. Surgery thus remains the most effective treatment for PPAS and AS-GCT. This is in keeping with extra-gonadal angiosarcomas, which also appear to be resistant to chemotherapies and radiotherapy [5, 15, 23]. Radical orchidectomy is also the mainstay surgical treatment for GCTs [8, 9, 26]. Platinum-based chemotherapy is the standard treatment for testicular germ cell tumours with heightened potential for metastasis or proven pre-existing metastasis [9, 27]. Teratomas are highly sensitive to cisplatin-based chemotherapy [9, 27]. Seminomatous metastases are particularly responsive to radiotherapy [28–30]. In general, testicular germ cell tumours are associated with excellent prognoses, even in the presence of metastatic disease [8–10].

Overall, two (40%) of the five PPAS cases suffered metastatic disease and died shortly thereafter, whereas three (60%) were alive without metastasis at follow-up, including the index case (Table 2). In contrast, only two (25%) of the eight cases of AS-GCT were alive without metastasis at follow-up and two (25%) had died (Table 4).

While follow-up was limited for most cases, survival for gonadal PPAS appeared superior to primary angiosarcoma that arises in extra-gonadal sites [1, 4, 15]. This is in keeping with a previous study that noted differential outcomes between testicular (gonadal) and mediastinal germ cell tumours (extra-gonadal) with sarcomatous transformation [15]. This might be because testicular angiosarcomas present earlier due to their location, ie easy access to self-examination, frequently prior to metastasis, and are amenable to complete resection using radical orchidectomy, compared to extra-gonadal angiosarcomas [4, 15].

In conclusion, PPAS is a rare testicular neoplasm. We present the fifth case in the literature. Clinical and radiological features are non-specific. The diagnosis is purely histological, with the pathologist choosing immunohistochemistry based on abnormal tumour morphology. Local invasiveness is variable but metastatic sites are typical for extra-gonadal angiosarcomas. PPAS confers a relatively better prognosis as compared to AS-GCT. While extra-gonadal angiosarcomas are associated with high rates of local recurrence following resection, in all cases of PPAS and AS-GCT there were no local recurrences following radical orchidectomy. Chemotherapy is associated with poor outcomes, thus surgical resection remains the most effective treatment for both subtypes of testicular angiosarcoma.

## Data Availability

The datasets generated and/or analysed during the current study are not publicly available due to patient confidentiality but are available from the corresponding author on reasonable request, pending patient consent.

## Abbreviations

AFP: Alpha-fetoprotein
AS: Angiosarcoma
AS-GCT: Angiosarcoma associated with germ cell tumour
CRP: C-reactive protein
CT: Chemotherapy
ESR: Erythrocyte sedimentation rate
LDH: Lactate dehydrogenase
LNs: Lymph nodes
MDT: Multi-disciplinary team
Met: Metastasis
MT: Mature teratoma
MTX: Methotrexate
N/A: Not communicated in original case report
PPAS: Primary pure angiosarcoma
RPLND: Retroperitoneal lymph node dissection
RT: Radiotherapy
β-HCG: Beta-human chorionic gonadotrophin
